# Design, Synthesis, Physicochemical Properties, and Biological Activity of Thymidine Compounds Attached to 5,8-Quinolinedione Derivatives as Potent DT-Diaphorase Substrates

**DOI:** 10.3390/ijms252011211

**Published:** 2024-10-18

**Authors:** Monika Kadela-Tomanek

**Affiliations:** Department of Organic Chemistry, Faculty of Pharmaceutical Sciences in Sosnowiec, Medical University of Silesia, 4 Jagiellońska Str., 41-200 Sosnowiec, Poland; mkadela@sum.edu.pl; Tel.: +48-323641666

**Keywords:** 5,8-quinolinedione, thymidine, AZT, NQO1

## Abstract

After heart disease, cancer is the second-leading cause of death worldwide. The most effective method of cancer treatment is target therapy. One of the potential goals of therapy could be DT-diaphorase, which reduces quinone moiety to hydroquinone, and reactive oxygen species are create as a byproduct. The obtaining of hybrid compounds containing the quinone moiety and other bioactive compounds leads to new derivatives which can activate DT-diaphorase. The aim of this research was the synthesis and characterization of new hybrids of 5,8-quinolinedione with thymidine derivatives. The analysis of the physicochemical properties shows a strong relationship between the structure and properties of the tested compounds. The enzymatic assay shows that hybrids are good substrates of NQO1 protein. The analysis of the structure–activity relationship shows that the localization of nitrogen atoms influences the enzymatic conversion rate. The analysis was supplemented by a molecular docking study. Comparing the results of the enzymatic assay and the molecular docking presents a strong correlation between the enzymatic conversion rate and the scoring value.

## 1. Introduction

Cancer is one of the most important health problems worldwide. One of the most frequently employed methods of cancer treatment is chemotherapy, which is used as a primary treatment method or as a complementary therapy after surgery or radiotherapy [1,2,3]. The potential goal of therapy can be DT-diaphorase (NQO1), which occurs at high levels in many types of human cancer, such as lung, breast, skin, and head and neck cancer. NQO1 is a flavoprotein catalysing the two-electron reduction of quinone to hydroquinone using the electron donor, such as nicotinamide adenine dinucleotide (NADP) or nicotinamide adenine dinucleotide phosphate (NADPH). The activation of protein causes the formation of reactive oxygen species (ROS), which causes DNA mutation and cell apoptosis [4,5].

**Figure 1:**
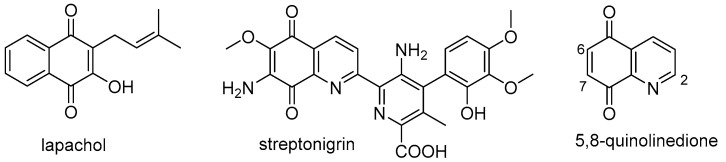
Chemical structure of a compound with quinone moiety.

The inspiration to create new synthetic compounds includes natural substances which are isolated from plants, microorganisms, and some animals. Although natural substances constitute only 3.8% of used drugs, their semi-synthetic derivatives constitute over 18% of employed drugs [6]. The analysis of the structure–activity relationship (SAR) of natural substance leads to the identification of a leading moiety, whose modification could receive the derivatives with higher biological activity and low toxicity [7,8]. Lapachol and streptonigrin are natural compounds which contain 1,4-naphthoquinone and 5,8-quinolinedione moiety, respectively (Figure 1). Both compounds are characterized by cytotoxic properties against cancer cells, bacteria, and fungi. These substances have not been used in cancer treatment because they exhibit high toxicity against normal cell lines [9,10,11,12,13]. The SAR analysis shows that the biological properties of streptonigrin depends on the substituent in the 5,8-quinolinedione fragment (Figure 1) [14]. Comparing the activity of compounds with 5,8-quinolinedieone and 1,4-naphthoquinone shows that a nitrogen atom increase their activity [15,16]. The introduction of a simple substituent at the C2, C6, and C7 positions of these fragment allows for the production of a compound characterized by activity against cancer cell lines [17,18,19,20,21].

In recent years, studies in the literature have described a hybrid of 5,8-quinolinedione with natural compounds which exhibit anticancer activity against brain, melanoma, breast, and female reproductive system cancers [15,20,22].

Currently, nearly twenty nucleosides drug are used in the clinical treatment of cancer. The main mechanism of action of these drug causes an interaction with the DNA or RNA, leading to cell apoptosis [23]. Structural modification of the nucleosides by attaching other functional groups leads to the production of compounds exhibiting interesting anticancer activity. Studies regarding the mechanism of the biological action show that the nucleoside derivatives often interact with an enzyme other than the natural nucleoside. It is worth noting that the interaction of nucleosides and their derivatives with the NQO1 protein has not been previously described in the literature [24,25,26]. The most recognised nucleoside derivative is 3′-azido-3′-deoxythymidine (AZT), which is used in the treatment of human immunodeficiency virus (HIV). The literature described that AZT shows a synergistic effect with cisplatin, methotrexate, or 5-fluorouracil in the therapy for advanced colon cancer [27,28]. The compound and its derivatives show potential activity against breast, lung, and colorectal cancer cells [29,30].

The introduction of a new group to the key structure of a compound may lead to changes in the in vivo pharmacokinetics of drugs. Determining the parameters of absorption, distribution, metabolism, excretion, and toxicity (ADMET) before in vivo testing allows for the prediction of the influence of chemical modification on the organism. The in silico ADMET tools are often used as a first step in QSAR analysis. However, the low accuracy of in silico methods encourages the comparison of the results with those of experimental methods [31,32].

In this research, the hybrids of 5,8-quinolinedione with thymidine derivatives were synthesised. The combination of nucleoside with 5,8-quinolinedionone moiety allows for the production of a new class of hybrids, which are characterised by low toxicity and better bioavailability than streptonigrin. For this reason, the physicochemical properties of the compounds were determined using in silico tools. The obtained compounds were examined as substrates of NQO1 protein, and the interaction with the enzyme was examined using a molecular docking study.

## 2. Results

### 2.1. Chemistry

**Scheme 1:**
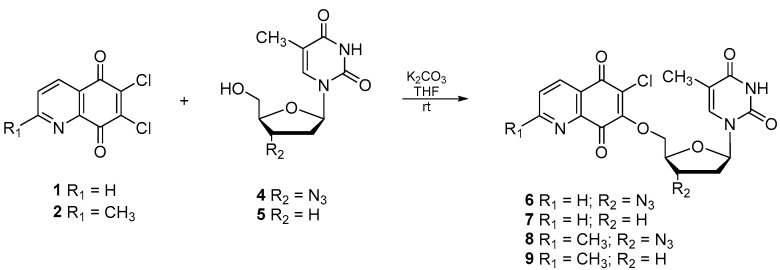
The synthesis routes of derivatives **6**–**9**.

The 6,7-dichloro-5,8-quinolinedione compounds 1–2 and 6,7-dichloro-5,8-isoquinolinedione 3 were obtained by the oxidation of 8-hydroxyquinoline [21,33]. The 5,8-quinolinedione 1–2 compounds were connected with thymidine derivatives, such as 3-azido-3-deoxythymidine 4 and 3-deoxythymidine 5, in the presence of potassium carbonate and tetrahydrofuran (Scheme 1). After purification, the hybrids 6–9 were obtained, with a 48–81% yield.

**Scheme 2:**
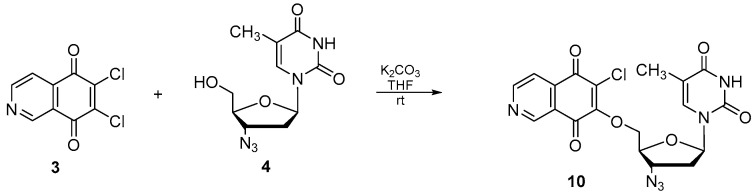
The synthesis routes of compound **10**.

The 5,8-isoquinolinedione hybrid with 3-azido-3-deoxythymidine 10 was obtained from the reaction between 6,7-dichloro-5,8-isoquinolinedione 3 and 3-azido-3-deoxythymidine 4, under the same conditions as those used for compounds 6–7 (Scheme 2).

The chemical structure of all synthesised compounds, such as ^1^H NMR, ^13^C NMR, and HR-MS, was confirmed using spectroscopic methods.

### 2.2. Molecular Descriptors

**Figure 2:**
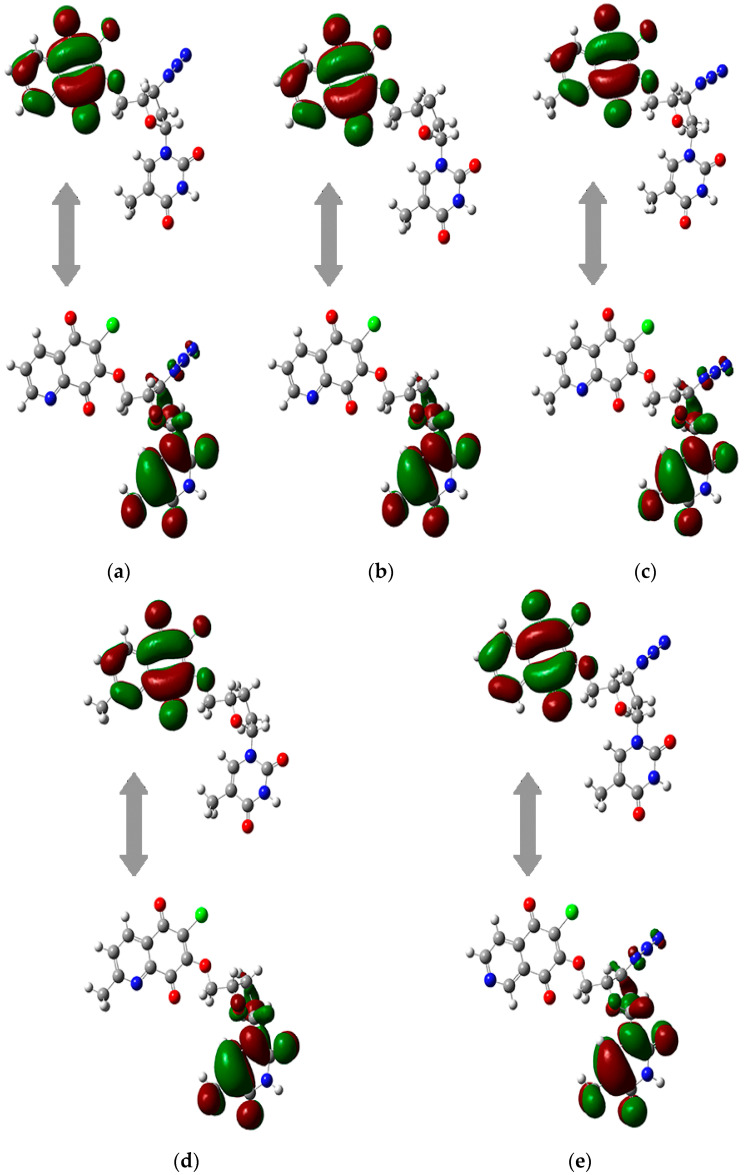
The HOMO and LUMO orbitals for compounds (**a**) 6; (**b**) 7; (**c**) 8; (**d**) 9; (**e**) 10.

Molecular descriptors refer to the physicochemical properties of a substance, which determine the reactivity and biodistribution of a compound. The first step was the optimalization of the molecular structure using the DFT (B3LYP/6-31G+(d.p)) method implemented in the Gaussian 16 program package [34]. The obtained structure was used to determine the highest occupied (HOMO) and lowest unoccupied (LUMO) molecular orbitals and to calculate their energy. The localization of the orbitals for all tested compounds is presented on Figure 2.

The energy gap (ΔE) between these orbitals allows for the determination of the ionization potential (I), electron affinity (A), hardness (η), chemical potential (µ), electronegativity (χ), and electrophilicity index (ω) [35]. The reactivity descriptors were calculated using the Gaussian 9.0 software [34,36] and are presented in Table 1.

**Figure 3:**
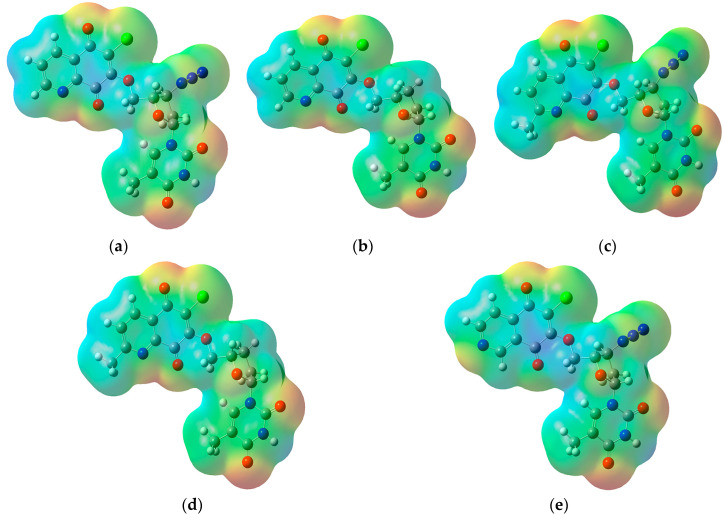
The molecular potential maps for compounds (**a**) 6; (**b**) 7; (**c**) 8; (**d**) 9; (**e**) **10**. The red, blue and green colours represent a nucleophilic, electrophilic and neutral regions, respectively.

The interaction of the compounds with other molecules depends on the arrangement of the nucleophilic and electrophilic regions. The molecular electrostatic potential map (MEP) allows for the visualization of different regions of the compound using colours. The red colour indicates a nucleophilic region, blue shows an electrophilic region, and green represent the neutral region of the ligand [37]. The MEPs are sketched in the order of (−1.45) to 1.45 kcal/mol. The maps of compounds 6–10 are presented in Figure 3.

The biodistribution is designated using the Lipinski, Veber, and ADMET parameters [38,39]. These descriptors were confirmed using online programs such as swissADME and pkCSM [40,41]. The selected bioavailability parameters are shown in Table 2.

### 2.3. Enzymatic Activity

The ability of a substance to act as a substrate of the DT-diaphorase (NQO1) protein was examined using the oxidation of NADPH to NADP+ method [20,42]. In this method, the incubation of the tested substances with NQO1 leads to the reduction of the 1,4-quinone moiety to the 1,4-dihydroxyquinoline unit, and the reactive oxygen species (ROS) are created as a byproduct. The concentration of ROS was measured using the amount of oxidized NADPH present.

**Figure 4:**
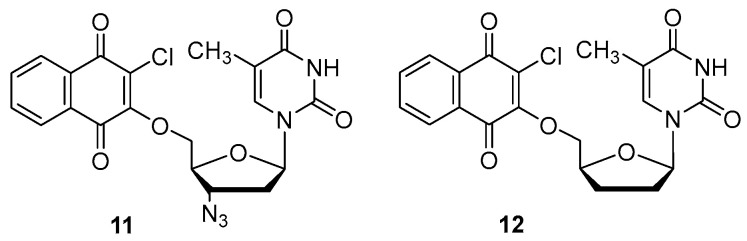
Chemical structure of compounds 11–12.

**Figure 5:**
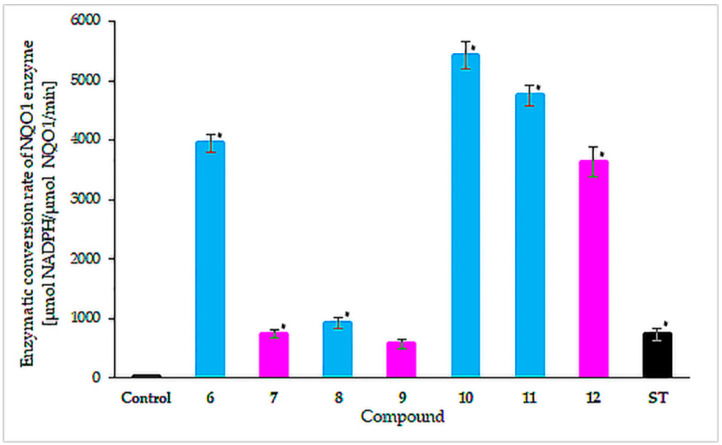
Enzymatic conversion rate of NQO1 for compounds 6–12 and streptonigrin (ST), monitored at an absorbance of A340 nm. Blue and pink colours indicate derivatives with 3-azido-3-deoxythymidine moiety and 3-deoxythymidine moiety, respectively. The black colour indicates reference substance. * *p* < 0.05 compared with the untreated control.

These studies used the compounds 6–10; 11–12, previously described hybrids with a 1,4-naphthoquinone moiety (Figure 4) [43]; and streptonigrin ST as a reference substance. The blank control contains DMSO instead of a compound. The obtained results for hybrids 6–12 and streptonigrin ST are presented as an enzymatic conversion rate of NOQ1 in Figure 5.

### 2.4. Molecular Docking

The interaction between the NQO1 protein and the ligands (1–3, 6–12, and ST) were examined using the molecular docking method. The results are presented as a scoring value (ΔG), which determines the binding energy between the protein and ligand. The lower value of ΔG means that the ligand is better fitted to the active centre of the protein. The results are shown in Table 3.

**Figure 6:**
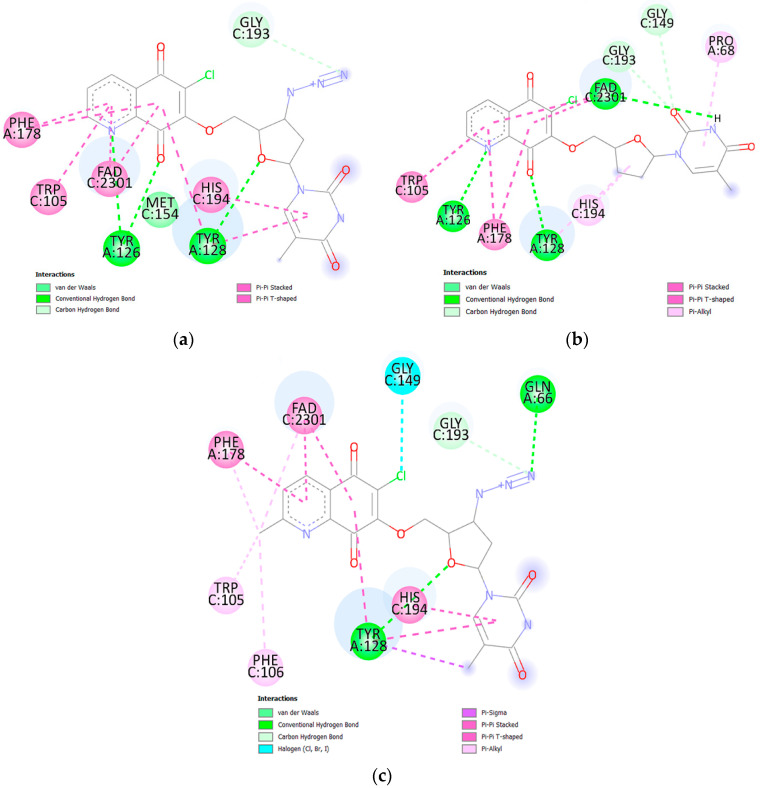
Docking pose of NQO1 protein complex with ligand (**a**) 6, (**b**) 7, (**c**) 8.

The detailed analysis of the interactions between the ligands and the proteins was carried out for ligands 6, 7, and 8 (Figure 6). The detailed data regarding the type and length of the binding interactions between these ligands and the protein residues are presented in Table S1.

## 3. Discussion

In the present study, five new compounds, which consist of 5,8-quinolinedione moiety and 3-deoxythymidine moiety, were obtained. Hybrids 6–10 were obtained with a high yield, and the structures were confirmed using spectroscopic methods (Figures S1–S5). For all compounds, the physicochemical properties determining the reactivity and bioavailability of the compounds were calculated.

The reactivity descriptors of the compounds are usually designated by the energy of the HOMO and LUMO orbitals. As seen on Figure 2, the HOMO orbital is mainly delocalized in the thymidine moiety. The energy of this orbital is similar for all tested structures, which means that it does not depend on the substituent at the C3 position of the thymidine moiety (Table 2). The LUMO orbital is delocalized near the 5,8-quinolinedione moiety (Figure 2), and the value of E_LUMO_ is in the range from −3.477 eV to −3.844 eV. The energy of the LUMO orbital depends on the type of 5,8-quinolinedione moiety; the order is as follows: 5,8-isoquinolinedione (10) > 5,8-quinolinedione (6 and 7) > 2-methyl-5,8-quinolinedione (8 and 9). The obtained parameters show that the compounds are characterized by kinetic stability and moderate chemical reactivity. Moreover, the tested compounds display an electrophilic character, and they are highly reactive with nucleophilic compounds [44,45]. The region of the compounds which interacts with the electrophilic and nucleophilic molecules are identified by using the molecular potential maps (MEP) [46,47]. As seen in Figure 3, the nucleophilic areas of compounds 6–10 are localized near the oxygen atom of the carbonyl group at the 5,8-quinolinedione and the thymidine moiety. For compounds 6 and 8, the additional nucleophilic area is localized near the azide substituent at C3 of the thymidine moiety. The blue colour indicates that the electrophilic areas are localized near the pyridine ring at the 5,8-quinolinedione moiety and the sugar moiety. The green coloured areas are localized near the thymidine ring and the chloride atom at the 5,8-quinolinedione moiety. The MEP analysis suggest that the 5,8-quinolinedione moiety is more reactive than the thymine moiety.

The analysis of the biodistribution parameters allow for the definition of the permeability of compounds across a biological barrier, like intestinal, skin, and blood–brain barriers. One of the most important parameters of substances with the potential to be used as drugs is water solubility. Compounds 6–10 are moderately solubilised in water, and the logS is in the range from −3.52 to −4.37. Hybrids with an azide substituent (6, 8, and 10) are characterized by better water solubility, with the inclusion of a hydrogen atom (7 and 9). Comparing the logS of derivatives 6, 8, and 10 shows that water solubility depends on the type of 5,8-quinolinedione moiety, following the order of 2-methyl-5,8-quinolinedione (8) > 5,8-quinolinedione (6) > 5,8-isoquinolinedione (10).

As seen in Table 2, the molecules 6–10 fulfil all Lipinski rules because the molecular weight is less than 500 g/mol, the logP is less than 5, and the number of acceptors (nHA) and donors (nHD) of hydrogen bonds is not more than 10 and 5, respectively. Comparing the Lipinski parameters of the compounds with those of the azide substituent (6, 8, and 10) and the hydrogen atom (7 and 9) at the C3 position of the sugar moiety shows that the substituent influences the lipophilicity and number of donors of the hydrogen bond (nHD). The analysis of the TPSA and nRT parameters shows that all derivatives also meet the Veber rule requirements. The results suggest that the compounds could exhibit good oral availability.

The ADMET parameters were determined in silico using the online-available software [40,41]. Human intestinal absorption (HIS) and Caco-2 permeability (logPapp) were used as the parameters for determining the adsorption of the orally administered drugs. For tested compounds **6**–**10**, the HIA and logPapp values are in the range of 61% to 72% and 0.185 to 0.849, respectively. The results suggest that hybrids 6–10 exhibit good absorption from the digestive system. The analysis of the relationship between absorption and the molecular structure of the compounds shows that the type of 5,8-quinolinedione moiety does not affect the Caco-2 permeability, while the introduction the azide group reduces the logPapp value. As seen in Table 2, derivatives 6–10 should be used as transdermal drugs because the logKp is lower than −2.5. The distribution of the compound was described by the volume of distribution (VDss), the permeability by blood–brain barrier (log BB), and the penetration to the central nervous system (log PS). The calculated VDss shows that the concentration of the compounds in the tissues is lower than in plasma. The derivatives with an azide substituent at the C3 position at the sugar moiety (6, 8, and 10) display a better distribution in the tissue than do compounds 7 and 8, containing a hydrogen atom at this position. The analysis of the log BB and log PS parameters shows that the tested compounds are poorly absorbed across the blood–brain barrier, and they do not penetrate to the central nervous system. The analysis shows that the derivatives should not be neurotoxic. The in silico metabolism of a drug is determined by the interaction of the substance with detoxification enzyme cytochrome P450. All derivatives 6–10 can be substrates of cytochrome P450 and will not inhibit its activity. The total clearance (CLtot) of 6–10 is in the range of 0.614 to 0.793. Comparing the value of CLtot shows a lower values for compounds with a hydrogen atom at the C3 position of the thymidine moiety (7 and 8) than for those with an azide group (6, 8 and 10). The compounds 6–10 are characterized by moderate toxicity, and the LD_50_ value is in the range of 2.088 to 2.311 mol/kg. The toxicity depends on the type of substituent at the C3 position of the thymidine moiety and do not depend on the type of 5,8-quinolinedione moiety.

The compounds containing the 1,4-quinone moiety interact with NQO1, which is overexpressed in many types of cancer, such as lung, brain, breast, and head and neck cancers [48,49]. My previous research shows that hybrids of 1,4-naphthoquinone with a thymidine moiety, such as 11–12, show activity against two squamous cell cancer lines (SCC-9 and SCC-25), which exhibit different expression levels of the gene encoding the NQO1 protein. Comparing the IC_50_ value against these cell lines shows that derivatives **11**–**12** exhibit almost 1.5-times more activity against SCC-9, which is characterized by the overexpression of the gene encoding the NQO1 protein [43]. Continuing my research, I tested the new compounds **6**–**10** and derivatives 11–12 as a substrate of NQO1 protein; streptonigrin (ST) was used as a reference. As seen in Figure 5, most of the tested compounds 6–12 are characterized by a higher enzymatic conversion rate than those of the reference substance. Compounds with a hydrogen atom at the C3 position of the thymidine moiety (7, 9, and 12) are characterized by a lower enzymatic conversion rate of NQO1 than those with an azide moiety at this position (6, 8, and 11). Moreover, the conversion rate depends on the type of 5,8-quinolinedione moiety, as follows: 5,8-isoquinoline (**10**) > 1,4-naphthoquinoline (11 and 12) > 5,8-quinolinedione (6 and 7) > 2-methyl-5,8-quinolinedione (8 and 9).

The next step in the research was to study the interaction between the protein and the ligand. As seen in Table 3, the scoring values of ligands 6–12 are higher than the ΔG of 6,7-dichloro-5,8-quinolinedione 1–3, and streptonigrin ST. Comparing the experimental and modelling results shows that hybrids with higher values of enzymatic conversion are characterized by a higher scoring value. The analysis of the binding energy of the ligand with different substituents at the C3 position of the thymidine moiety shows that the ligand with the azide group (6, 8, 10, and 11) are characterized by higher values than those with a hydrogen atom (7, 9, and 12).

As seen on Figure 6, the ligands 6 and 7 create a hydrogen bond and a hydrophobic interaction. For both NOQ1–ligands, the nitrogen and oxygen atoms of the 5,8-quinolinedione moiety create a hydrogen bond with TYR126 and TYR128, respectively. Moreover, the 5,8-quinolinedione creates a hydrophobic interaction with the TRP105, PHE188, and FAD cofactors. The introduction of the thymidine moiety at the C7 position of 5,8-quinolinedione does not influence the arrangement of 5,8-quinolinedione in the binding pocket of the enzyme (Figure S6). The arrangement of the thymidine moiety depends on the substituent at the C3 position. The 3-azido-3-deoxythymidine moiety in ligand **6** creates hydrogen bonds with GLY193 and TYR128, while the 3-deoxythymidine moiety in ligand **7** interacts via the H-bond with the GLY193, GLY149, and FAD cofactors. 

The enzymatic study shows that compounds 8 and 9, with the 2-methyl-5,8-quinolinedione moiety, exhibit low activity against the NQO1 enzyme. Comparing the arrangement of ligands with 2-methyl-5,8-quinolinedione (2 and 8) and with 5,8-quinolinedione (1 and 6) in the active site of the enzyme shows that the methyl group at the C2 position of the 5,8-quinolinedione moiety influences their interaction with the enzyme (Figure 6 and Figure S6). In both cases, the 5,8-quinolinedione moiety creates hydrophobic interactions (PHE178, TRP105, TYR128, and FAD cofactor) and halogen interactions between the chloride atom and GLY149. The arrangement of the thymidine moiety in the complex NQO1-ligand 8 is similar to that for ligand 6. The results suggest that the creation of a hydrogen bond is necessary to maintain high enzymatic activity.

The arrangement of ligands 6–8 in the active centre of the enzyme was compared with the calculated electrostatic potential map. The nucleophilic region of the compounds creates a hydrogen bond with the amino acid in the binding pocket of the enzyme, while the electrophilic region localized near the pyridine moiety creates the hydrophobic interaction. The electron neutral region near the thymidine moiety creates a weak hydrophobic interaction with HIS194. The analysis shows the electrostatic potential influence on the binding in the pocket of the NQO1 protein.

## 4. Materials and Methods

All commercial substance were purchased from Merck (Darmstadt, Germany) and were used without purification. The Bruker Avance 600 spectrometer (Bruker, Billerica, MA, USA) was used to measure the nuclear magnetic resonance (NMR) spectra. The compounds (10 mg) were dissolved in *d*6-acetone (0.6 mL). The chemical shift (δ) and coupling constant (*J*) are reported in ppm and Hz, respectively. The multiplicity is indicated using the following classifications: broad singlet (bs), singlet (s), doublet (d), doublet of doublets (dd), triplet (t), and multiplet (m). The Bruker Impact II instrument (Bruker, Billerica, MA, USA) was used to obtain the high-resolution mass spectra (HR-MS). The theoretical molecular mass of the compounds was calculated using The Exact Mass Calculator, Single Isotope Version (http://www.sisweb.com/referenc/tools/exactmass.htm, accessed on 12 October 2024; (Ringoes, NJ, USA).

### 4.1. Chemistry Study

The 5,8-quinolinedione compounds **1**–**3** (0.5 mmol) and potassium carbonate (2 eqv., 1.0 mmol, 0.138 g) were dissolved in tetrahydrofuran (4 mL). After 15 min, the thymidine derivatives **4**–**5** (2 eqv., 1 mmol), in tetrahydrofuran (1 mL), was added. The reaction progress was controlled by thin layer chromatography (TLC). After 24 h, the solvent was evaporated under reduced pressure. The dry residue was purified using column chromatography (CHCl_3_: EtOH, 15:1, *v*/*v*). After that, obtain pure compound **6**–**10** was obtained, as follows:

#### 4.1.1. 7-(3′-Azido-3′-deoxythymidine)-6-chloro-5,8-quinolinedione **6**

Yield 67%; oil; ^1^H NMR (600 MHz, *d*6-acetone) δ, ppm: 1.78 (s, H7t), 2.53 (m, 1H, H2′t), 2.73 (m, 1H, H2′t), 4.25 (m, 1H, H3′t), 4.77 (m, 1H, H4′t), 4.91 (dd, *J* = 3.6 Hz, *J* = 12.0 Hz, 1H, H5′t), 5.11 (dd, *J* = 3.0 Hz, *J* = 11.4 Hz, 1H, H5′t), 6.28 (t, *J* = 6.6 Hz, 1H, H1′t), 7.57 (s, 1H, H6t), 7.87 (dd, *J* = 7.8 Hz, *J* = 4.8 Hz, 1H, H3q), 8.46 (dd, *J* = 1.2 Hz, *J* = 7.8 Hz, 1H, H4q), 9.03 (dd, *J* = 1.8 Hz, *J* = 4.8 Hz, 1H, H2q), 9.98 (bs, 1H, NHt); ^13^C NMR (150 MHz, *d*6-acetone) δ, ppm: 11.7, 36.4, 60.5, 72.7, 82.5, 84.4, 110.3, 126.8, 128.2, 134.2, 135.7, 147.2, 154.5, 157.6, 163.2, 177.8; ESI-HRMS *m*/*z* [M + Na]^+^ calcd. for C_19_H_15_ClN_6_O_6_Na^+^ 481.0639, found 481.0600.

#### 4.1.2. 6-Chloro-7-(3′-deoxythymidine)-5,8-quinolinedione **7**

Yield 53%; oil; ^1^H NMR (600 MHz, *d*6-acetone) δ, ppm: 1.79 (d, *J* = 1.2 Hz, 3H, H7t), 2.23 (m, 3H, H2′t, 2 × H3′t), 2.42 (m, 1H, H2′t), 4.42 (m, 1H, H4′t), 4.81 (dd, *J* = 3.6 Hz, *J* = 12.0 Hz, 1H, H5′t), 5.08 (dd, *J* = 3.6 Hz, *J* = 12.0 Hz, 1H, H5′t), 6.11 (t, *J* = 6.6 Hz, 1H, H1′t), 7.55 (d, *J* = 1.2 Hz, 1H, H6t), 7.86 (dd, *J* = 7.8 Hz, *J* = 4.8 Hz, 1H, H3q), 8.45 (dd, *J* = 1.2 Hz, *J* = 7.8 Hz, 1H, H4q), 9.02 (dd, *J* = 1.8 Hz, *J* = 4.8 Hz, 1H, H2q), 9.87 (bs, 1H, NHt); ^13^C NMR (150 MHz, *d*6-acetone) δ, ppm: 11.7, 25.5, 30.8, 74.3, 78.9, 85.3, 109.8, 126.6, 128.0, 134.1, 135.7, 147.2, 154.4, 158.1, 163.3, 177.8; ESI-HRMS *m*/*z* [M + Na]^+^ calcd. for C_19_H_16_ClN_3_O_6_Na^+^ 440.0625, found 440.0615.

#### 4.1.3. 7-(3′-Azido-3′-deoxythymidine)-6-chloro-2-methyl-5,8-quinolinedione **8**

Yield 81%; oil; ^1^H NMR (600 MHz, *d*6-acetone) δ, ppm: 1.78 (s, H7t), 2.53 (m, 1H, H2′t), 2.70 (s, 3H, CH_3_), 2.73 (m, 1H, H2′t), 4.24 (m, 1H, H3′t), 4.76 (m, 1H, H4′t), 4.87 (dd, *J* = 3.6 Hz, *J* = 12.0 Hz, 1H, H5′t), 5.10 (dd, *J* = 3.0 Hz, *J* = 11.4 Hz, 1H, H5′t), 6.27 (t, *J* = 6.6 Hz, 1H, H1′t), 7.57 (s, 1H, H6t), 7.72 (d, *J* = 7.8 Hz, 1H, H3q), 8.32 (d, *J* = 1.2 Hz, 1H, H4q), 9.98 (bs, 1H, NHt); ^13^C NMR (150 MHz, *d*6-acetone) δ, ppm: 11.6, 24.0, 36.3, 60.4, 72.5, 82.4, 84.4, 110.3, 126.1, 126.7, 127.4, 127.6, 134.4, 135.7, 146.5, 150.2, 157.4, 164.7, 177.8; ESI-HRMS *m*/*z* [M + Na]^+^ calcd for C_20_H_17_ClN_6_O_6_Na^+^ 495.0795, found 495.0779.

#### 4.1.4. 6-Chloro-7-(3′-deoxythymidine)-2-methyl-5,8-quinolinedione **9**

Yield 48%; oil; ^1^H NMR (600 MHz, *d*6-acetone) δ, ppm: 1.79 (d, *J* = 1.2 Hz, 3H, H7t), 2.21 (m, 3H, H2′t, 2xH3′t), 2.43 (m, 1H, H2′t), 4.41 (m, 1H, H4′t), 4.77 (dd, *J* = 3.6 Hz, *J* = 12.0 Hz, 1H, H5′t), 5.08 (dd, *J* = 3.6 Hz, *J* = 12.0 Hz, 1H, H5′t), 6.11 (t, *J* = 6.6 Hz, 1H, H1′t), 7.53 (d, *J* = 1.2 Hz, 1H, H6t), 7.71 (d, *J* = 7.8 Hz, 1H, H3q), 8.30 (d, *J* = 1.2 Hz, 1H, H4q), 9.86 (bs, 1H, NHt); ^13^C NMR (150 MHz, *d*6-acetone) δ, ppm: 11.7, 24.1, 25.5, 30.7, 40.5, 74.0, 79.2, 85.3, 109.8, 126.0, 127.4, 134.3, 135.6, 146.7, 150.2, 157.9, 164.6, 177.7; ESI-HRMS *m*/*z* [M + Na]^+^ calcd for C_20_H_18_ClN_3_O_6_Na^+^ 454.0781, found 454.0775.

#### 4.1.5. 7-(3′-Azido-3′-deoxythymidine)-6-chloro-5,8-isoquinolinedione **10**

Yield 77%; oil; ^1^H NMR (600 MHz, *d*6-acetone) δ, ppm: 1.78 (s, H7t), 2.53 (m, 1H, H2′t), 2.74 (m, 1H, H2′t), 4.24 (m, 1H, H3′t), 4.75 (m, 1H, H4′t), 4.88 (dd, *J* = 3.6 Hz, *J* = 12.0 Hz, 1H, H5′t), 5.15 (dd, *J* = 3.0 Hz, *J* = 11.4 Hz, 1H, H5′t), 6.27 (t, *J* = 6.6 Hz, 1H, H1′t), 7.56 (m, 1H, H6t), 7.94 (dd, *J* = 0.6 Hz, *J* = 4.8 Hz, 1H, H3q), 9.12 (dd, *J* = 7.2 Hz, *J* = 4.8 Hz, 1H, H4q) 9.25 (d, *J* = 9.7 Hz, 1H, H1q), 9.97 (bs, 1H, NHt);^13^C NMR (150 MHz, *d*6-acetone) δ, ppm: 11.6, 36.3, 60.3, 72.6, 82.4, 84.5, 110.3, 124.0, 124.5, 127.7, 128.0, 135.7, 147.9, 150.3, 155.9, 157.0, 163.1, 177.7, 179.3; ESI-HRMS *m*/*z* [M + Na]^+^ calcd for C_19_H_15_ClN_6_O_6_Na^+^ 481.0639, found 481.0615.

### 4.2. Computational Details

The optimized chemical structure of compounds **6**–**10** was calculated using the density functional theory (DFT) method implemented in the Gaussian 16 program package [34]. The B3LYP exchange-correlation functional with 6-31+G(d.p) Gaussian basis set was applied. In our calculations, I applied the basis set with the diffuse function on heavy atoms (+) to obtain a better description of the lone pair electrons with orbitals occupying a larger region of space. All of the local minima of energy were confirmed by the absence of an imaginary mode in the vibrational calculations. All obtained results were visualized using the GaussView, version 5, software package [36].

### 4.3. Enzymatic Study

The enzymatic assay was determined using the previously described method [50,51,52]. Briefly, the DT-diaphorase (NQO1, EC 1.6.5.5, human recombinant, Sigma-Aldrich, St. Louis, MO, USA), in a 50 mmol/L potassium phosphate buffer (pH = 7.4), was added to a 96-well plate (Nunc Thermo Fisher Scientific, Waltham, MA, USA). Then, 2 µL of the compound solution (10 µmol/L) in the dimethyl sulfoxide (DMSO) was added to each well. After 2 min, an enzymatic reaction was initiated by adding the nicotinamide adenine dinucleotide phosphate (NADPH) (400 µmol/L) into each well. The conversion of NADPH to NADP^+^ was measured at the absorption wavelength of 340 nm using the BioTek 800TS microplate reader (BioKom, Janki, Poland). The data were recorded at 10 s intervals for 10 min at 25 °C. The NADPH oxidation rates were compared with the reactions of the samples lacking the compound. The initial velocities were calculated, and the data were expressed as µmol NADPH/µmol NQO1/min. All reactions were carried out at least in triplicate.

### 4.4. Molecular Docking Study

The crystal structure of DT-diaphorase (NQO1), identifier 2F1O, was downloaded from the Protein Data Bank (PDB) [52]. In the active site, the protein contains the flavin adenine dinucleotide (FAD) molecule as a cofactor. The structure of ligands **6**–**12** was optimized using the Gaussian software [34]. AutoDock Vina 1.5.6 software was used to perform the molecular docking study [53]. The grid centre of the Vina docking was selected as the centre of the reference ligands, which accompanied the downloaded protein complexes (x = 11.456160; y = 12.047880; z = −5.676920). The grid size was set to 15 Å × 15 Å × 15 Å, which is large enough to cover the entire target active site. Default values of all other parameters were used, and the complexes were subjected to eight genetic algorithm runs. All obtained results were visualized using the BIOVIA Discovery Studio 2021 software package [54].

## 5. Conclusions

In this study, new thymidine-5,8-quinolinedione hybrids were synthesized and characterized, and the physicochemical properties and the activity of the compounds as substrates of the NQO1 protein were determined. The analysis of the reactivity descriptor shows that the obtained hybrids are characterized by kinetic stability and moderate chemical reactivity. Moreover, they exhibit an electrophilic character. The molecular potential maps show that the nucleophilic areas are localized near the oxygen atom of the carbonyl groups, while the electrophilic areas are delocalized in the pyridine and sugar moieties. The calculated ADMET parameters show that the compounds could be used as orally administered drugs. The enzymatic assay shows that the localization of the nitrogen atom in the 5,8-quinolinedione moiety influences the enzymatic conversion rate. The introduction of a methyl group at the C2 position of the 5,8-quinolinedione moiety reduces the enzymatic activity. Comparing the results of the enzymatic assay and the molecular docking study shows a strong correlation between the enzymatic conversion rate and the scoring value. The docking results show that the introduction of a methyl group at the C2 position of 5,8-quinolinedione strongly influences the arrangement of ligands in the active site of the enzyme. Comparing the MEPs and their arrangement in the active site of the enzyme shows that the electrostatic potential influences the binding in the pocket of the NQO1 protein. It is worth noting that the study of new hybrids of thymidine as substrates for NQO1 protein may lead to new medical therapeutic applications in the future.

## Figures and Tables

**Table 1:** The reactivity descriptors of compounds **6–10**.

Compound	6	7	8	9	10
E_HOMO_ [eV]	−6.516	−6.434	−6.406	−6.417	−6.477
E_LUMO_ [eV]	−3.669	−3.593	−3.580	−3.477	−3.844
ΔE [eV]	2.847	2.841	2.826	2.940	2.634
I [eV]	6.516	6.434	6.406	6.417	6.477
A [eV]	3.669	3.593	3.580	3.477	3.844
η [eV]	1.423	1.420	1.413	1.470	1.317
µ [eV]	−5.093	−5.013	−4.993	−4.947	−5.160
χ [eV]	5.093	5.013	4.993	4.947	5.160
ω [eV]	9.110	8.848	8.821	8.323	10.112

**Table 2:** The biodistribution parameters of compounds 6–10.

Compound	6	7	8	9	10
logS	−4.04	−3.52	−4.37	−3.85	−3.84
M [g/mol]	458.8	417.8	472.8	431.8	458.8
TPSA [Å]	170.1	120.3	170.1	120.3	170.1
logP	2.09	1.81	2.37	1.86	2.24
nHA	10	7	10	7	10
nHD	1	1	1	1	1
nRT	5	4	5	4	5
logPapp [cm/s]	0.208	0.849	0.185	0.787	0.22
HIA [%]	61	63	62	63	72
logKp	−2.742	−2.775	−2.744	−2.784	−2.746
logBB	−1.909	−1.291	−1.926	−1.307	−1.913
logPS	−3.619	−3.499	−3.601	−3.482	−3.619
CYP3A4 substrate	yes	yes	yes	yes	yes
CYP3A4 inhibitor	no	no	no	no	no
CLtot [ml/min/kg]	0.793	0.703	0.750	0.614	0.789
LD_50_ [mol/kg]	2.073	2.297	2.088	2.311	2.067

**Table 3:** The energy scoring (ΔG) between the NQO1 protein and the ligands (6–12 and ST).

Ligand	ΔG [kcal/mol]	Ligand	ΔG [kcal/mol]
1	−8.1	9	−8.2
2	−7.8	10	−10.0
3	−8.1	11	−9.5
6	−9.4	12	−8.6
7	−8.9	ST	−7.1
8	−8.6		

## Data Availability

The data presented in the study are availability from the author.

## Supplementary Materials

The following supporting information can be downloaded at: https://www.mdpi.com/article/10.3390/ijms252011211/s1.
