# Supplementary material for: Design, Synthesis, Physicochemical Properties, and Biological Activity of Thymidine Compounds Attached to 5,8-Quinolinedione Derivatives as Potent DT-Diaphorase Substrates
Source: Int J Mol Sci. 2024 Oct 18;25(20):11211. doi: 10.3390/ijms252011211 (PMC11508761; doi:10.3390/ijms252011211)
Supplement: Supplementary file 1 [file ijms-25-11211-s001.zip › ijms-3235102-supplementary.pdf]

Design, Synthesis, Physicochemical Properties, and Biological Activity of Thymidine Compounds Attached to 5,8-Quinolinedione Derivatives as Potent DT-Diaphorase Substrates

Monika Kadela-Tomanek

Department of Organic Chemistry, Faculty of Pharmaceutical Sciences in Sosnowiec, Medical University of Silesia, 4 Jagiello ´nska Str., 41-200 Sosnowiec, Poland; mkadela@sum.edu.pl; Tel.: +48-323641666

Table of context

Table S1. Interaction of selected hybrids with active site of NQO1 protein.
Figure S1. Spectra of 7-(3′-azido-3′-deoxythymidine)-6-chloro-5,8-quinolinedione **6**
Figure S2. Spectra of 7-(3′-deoxythymidine)-6-chloro-5,8-quinolinedione **7**
Figure S3. Spectra of 7-(3′-azido-3′-deoxythymidine)-6-chloro-2-methyl-5,8-quinolinedione **8**
Figure S4. Spectra of 7-(3′-deoxythymidine)-6-chloro-2-metyl-5,8-quinolinedione **9**
Figure S5. Spectra of 7-(3′-azido-3′-deoxythymidine)-6-chloro-5,8-isoquinolinedione **10**
Figure S6. Docking pose of NQO1 protein complex with ligand (**a**) **1**; (**b**) **2**.

Table S1. Interaction of selected hybrids with active site of NQO1 protein.

Ligand	H-bonding residues and length (Å)	π-interaction residues and length (Å)	Halogen interaction residues and length (Å)
**6**	TYR126 (3.39, 3.35)<br>TYR128 (2.67)<br>GLY193 (3.58)	TRP105 (4.71, 5.35)<br>TYR128 (4.09, 5.21)<br>PHE178 (5.88)<br>HIS194 (4.71)<br>FAD (3.65, 4.02, 4.11, 3.96, 5.21)	
**7**	TYR126 (3.29)<br>TYR128 (2.71)<br>GLY149 (3.48)<br>GLY193 (3.54)<br>FAD (2.43)	PRO66 (5.04)<br>TRP105 (5.18, 4.57)<br>TYR128 (4.70)<br>PHE178 (5.39, 4.81)<br>HIS194 (4.44)<br>FAD (3.57, 4.07, 4.37, 3.90, 5.07)	
**8**	GLN66 (3.27)<br>TYR128 (3.33)<br>GLY193 (3.33)	TRP105 (4.66, 3.74)<br>PHE106 (4.56)<br>TYR128 (3.76, 4.62, 4.82)<br>PHE178 (4.60, 4.07)<br>HIS194 (4.41)<br>FAD (4.05, 3.66, 4.80, 4.65, 4.64, 4.81)	GLY149 (3.13)

Figure S1. Spectra of 7-(3′-azido-3′-deoxythymidine)-6-chloro-5,8-quinolinedione **6**

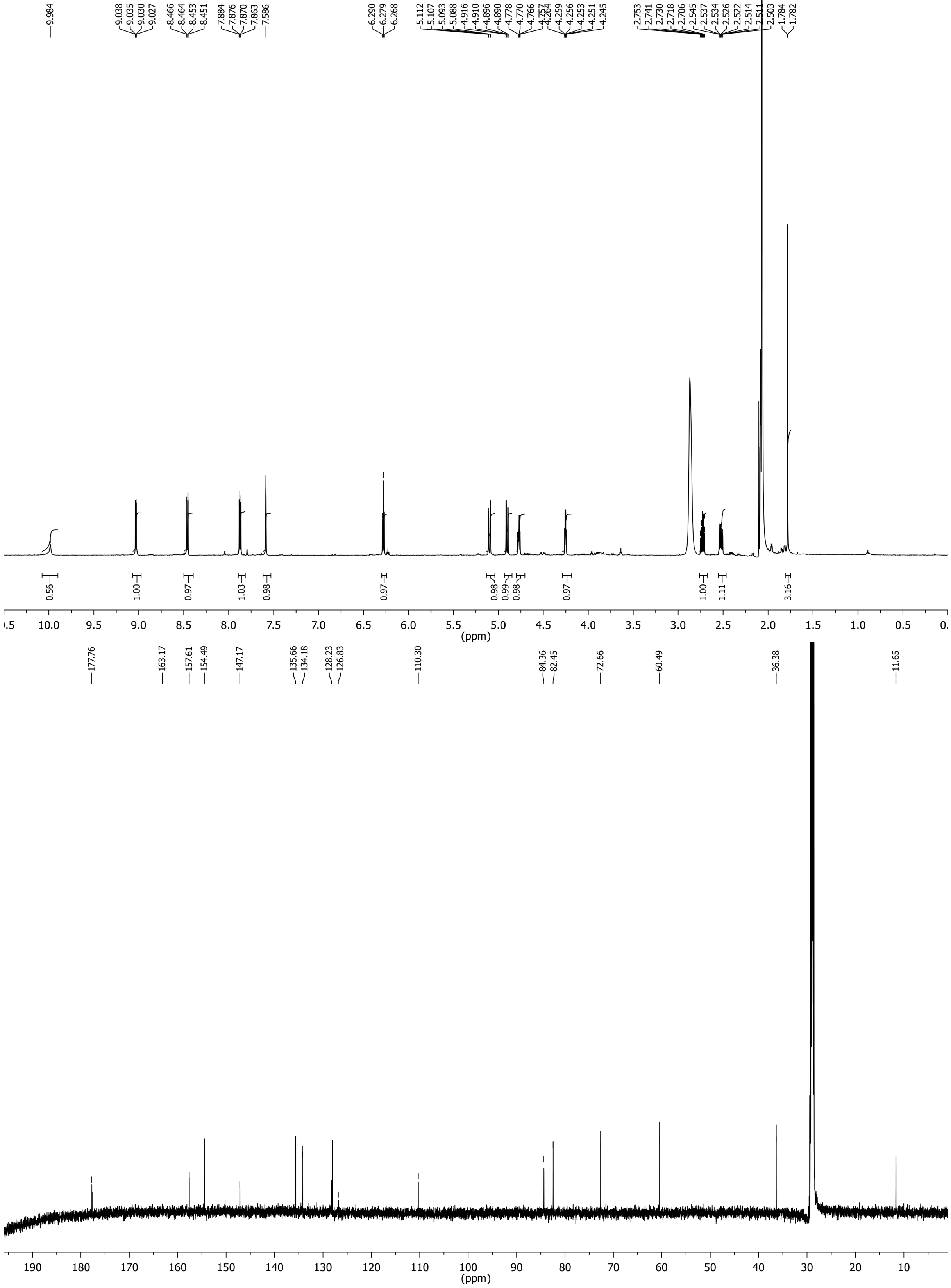

Figure S2. Spectra of 7-(3′-deoxythymidine)-6-chloro-5,8-quinolinedione **7**

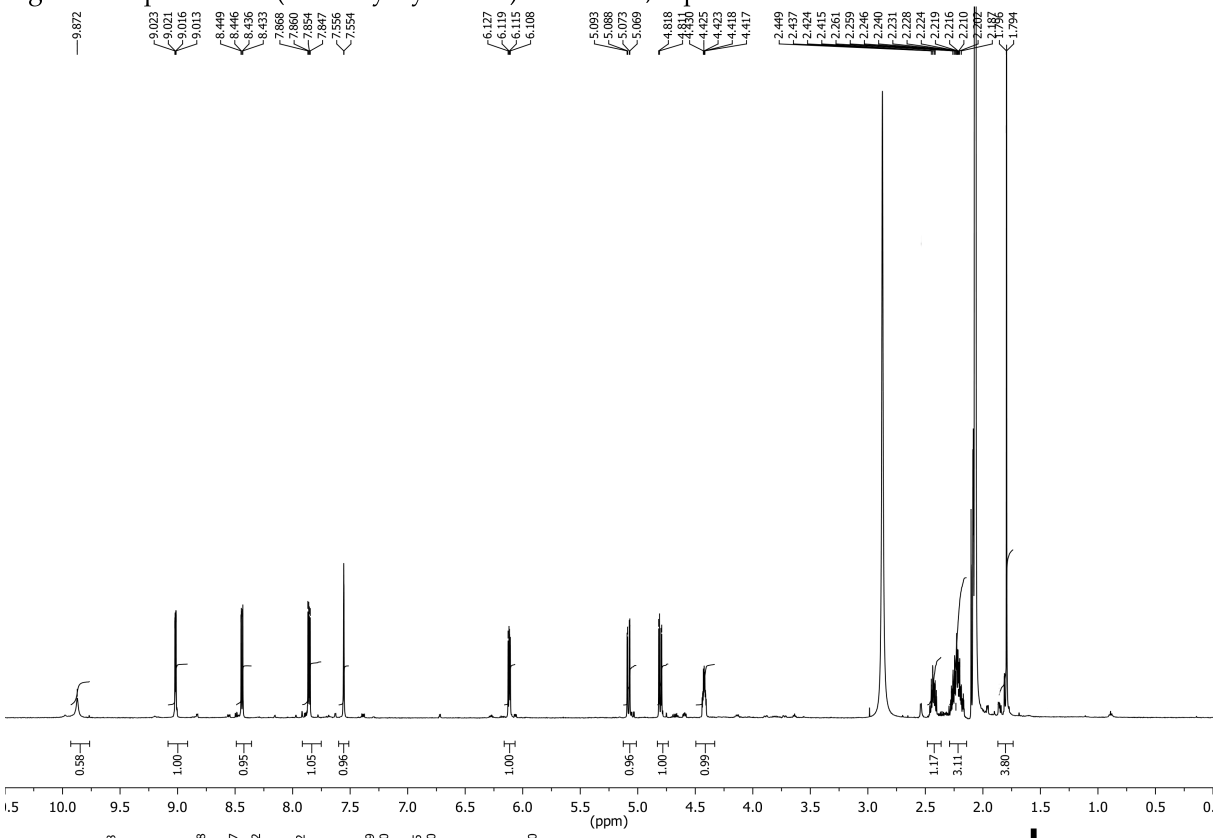

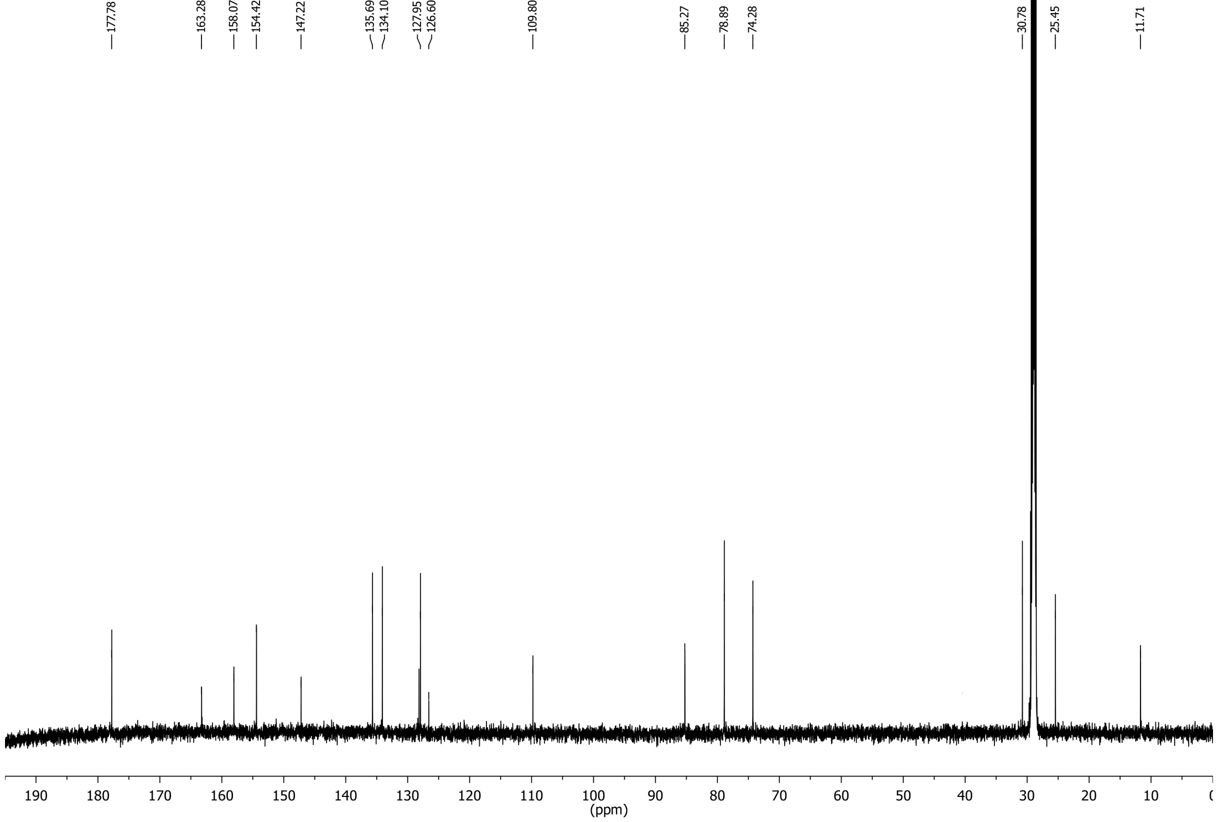

Figure S3. Spectra of 7-(3′-azido-3′-deoxythymidine)-6-chloro-2-methyl-5,8-quinolinedione **8**

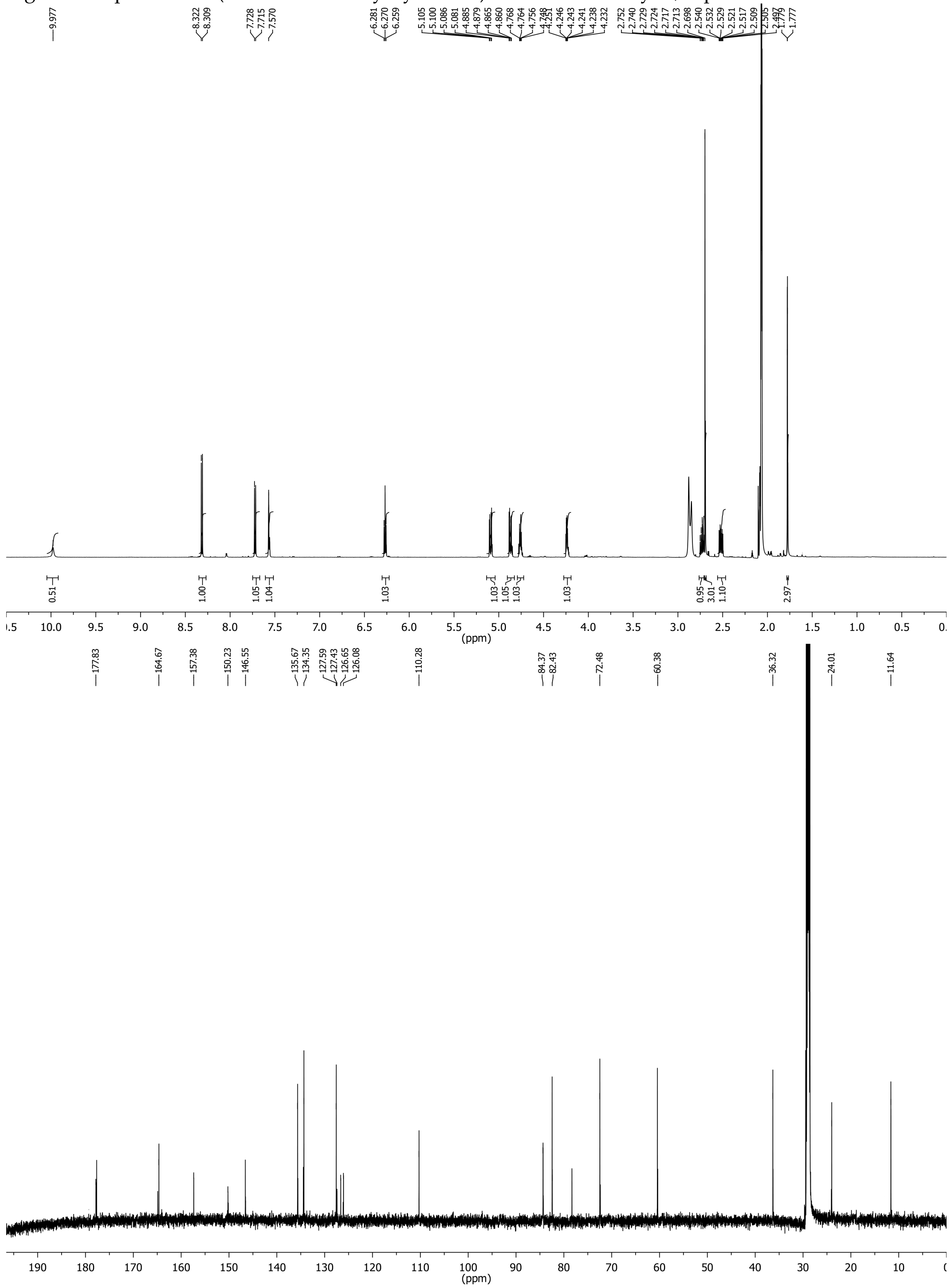

Figure S4. Spectra of 7-(3′-deoxythymidine)-6-chloro-2-metyl-5,8-quinolinedione **9**

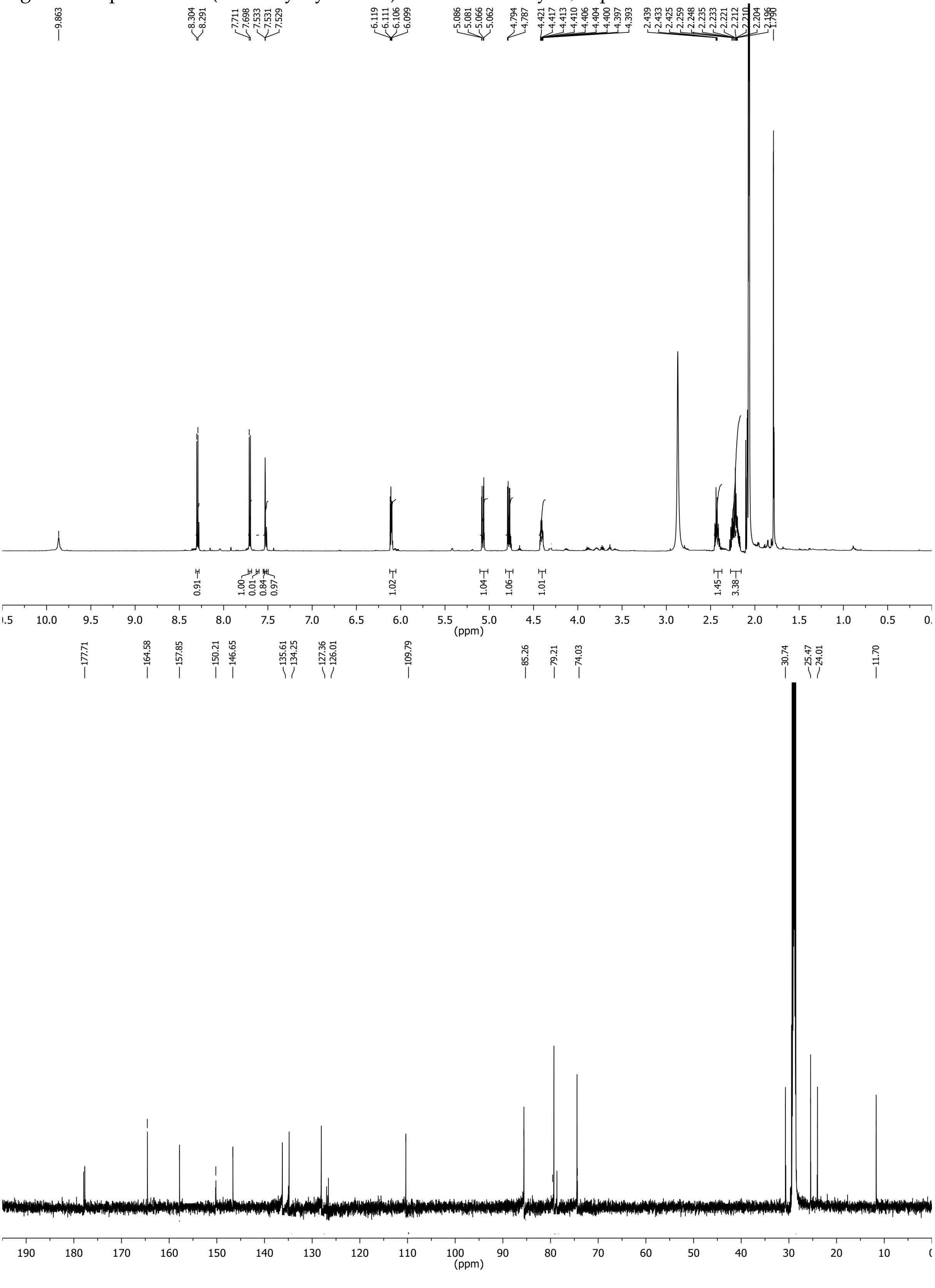

Figure S5. Spectra of 7-(3′-azido-3′-deoxythymidine)-6-chloro-5,8-isoquinolinedione **10**

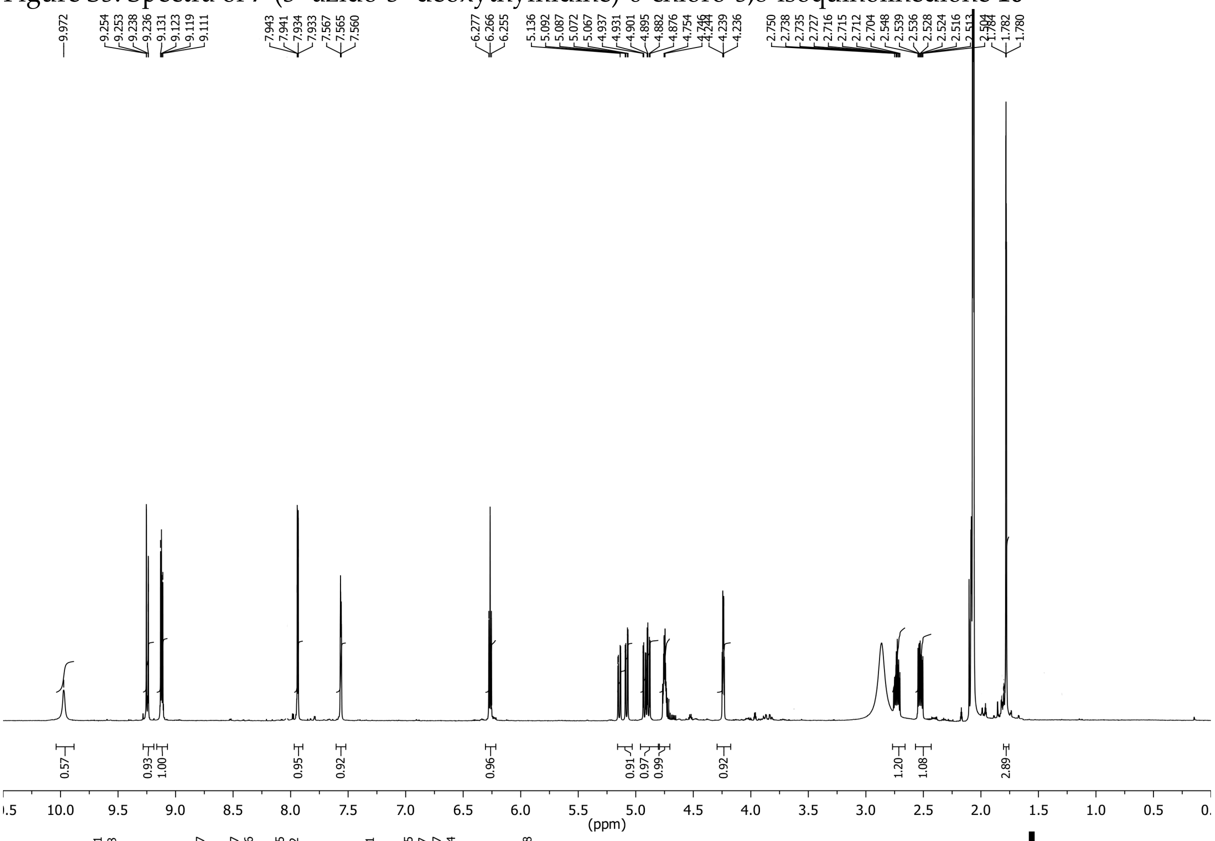

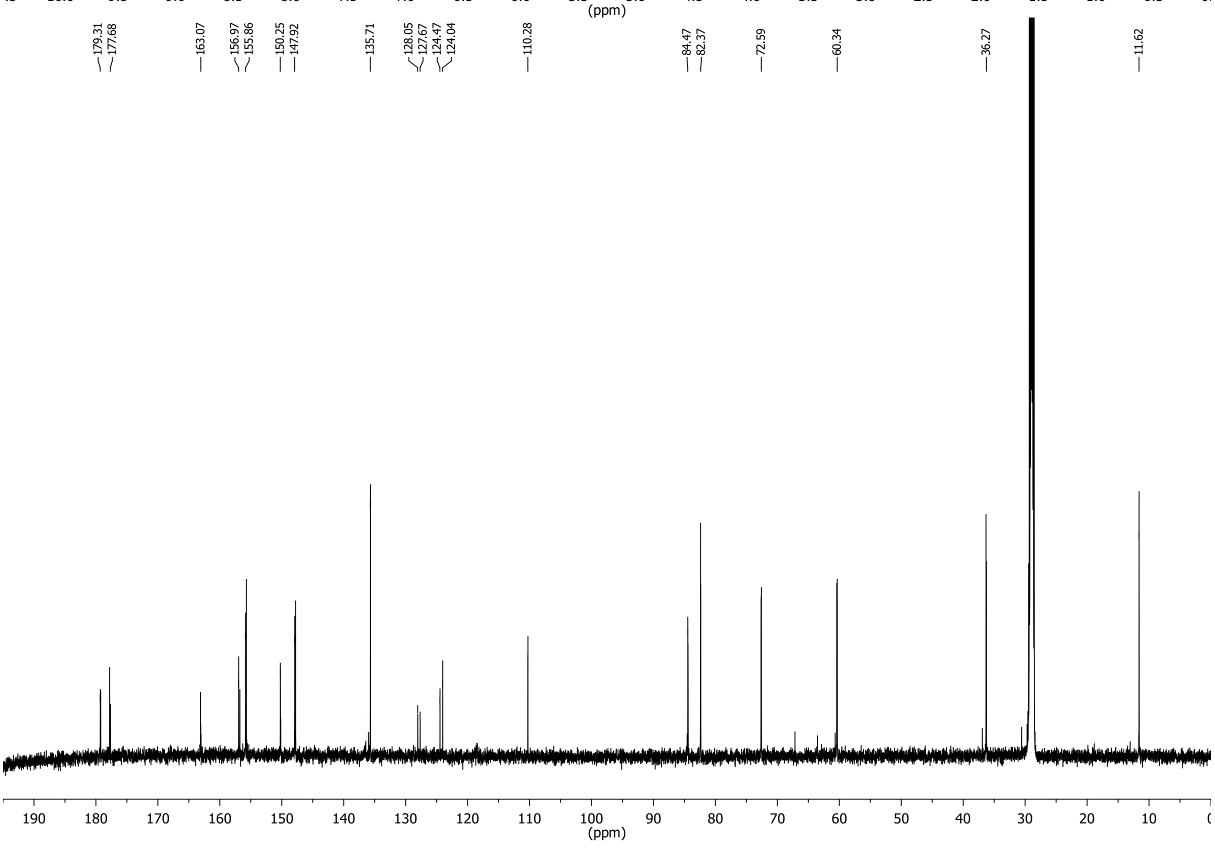

Figure S6. Docking pose of NQO1 protein complex with ligand (**a**) **1**; (**b**) **2**.

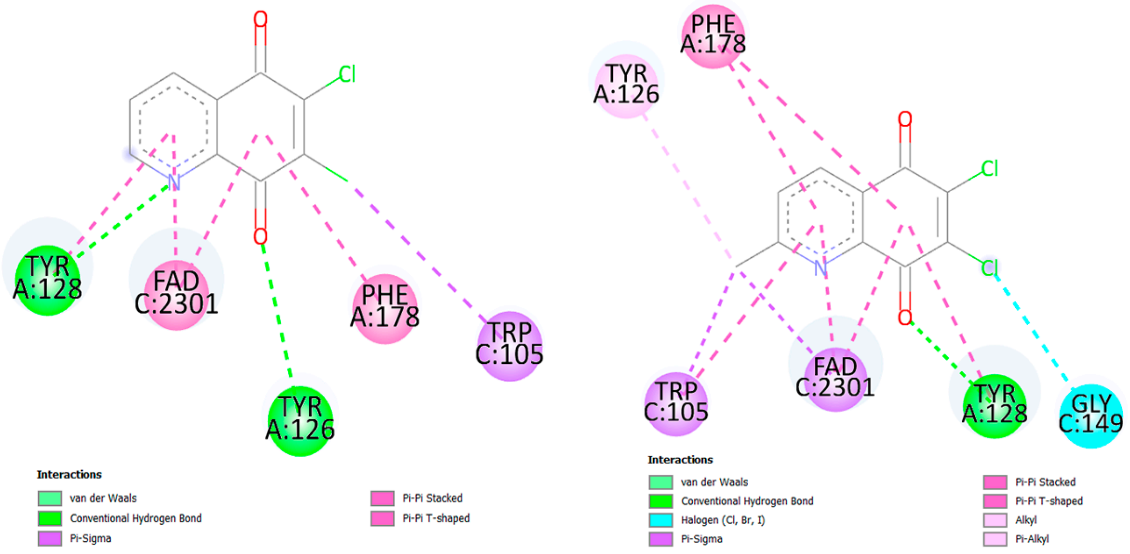

(**a**) (**b**)
